# Ultrasonic-Assisted Diels–Alder Reaction Exfoliation of Graphite into Graphene with High Resveratrol Adsorption Capacity

**DOI:** 10.3390/nano11113060

**Published:** 2021-11-14

**Authors:** Jinxing Zhang, Qi Ouyang, Qilin Gui, Xiaonong Chen

**Affiliations:** Beijing Laboratory of Biomedical Materials, Beijing University of Chemical Technology, Beijing 100029, China; zhangjinxing1202@163.com (J.Z.); ouyangqi0000@163.com (Q.O.)

**Keywords:** graphene, Diels–Alder reaction, exfoliation, resveratrol, adsorption

## Abstract

Scalable preparation of graphene with high adsorption capacity is an important prerequisite for fully realizing its commercial application. Herein, we propose an environmentally friendly route for exfoliation of graphene, which is established based on the Diels–Alder reaction. In our route, *N*-(4-hydroxyl phenyl) maleimide enters between the flakes as an intercalating agent and participates in the Diels–Alder reaction as a dienophile to increase the interlayer spacing of graphite. Then, graphite is exfoliated into graphene with the aid of ultrasound. The exfoliated product is hydroxyl phenyl functionalized graphene with a thickness of 0.5–1.5 nm and an average lateral size of about 500–800 nm. The exfoliation process does not involve any acid or catalyst and would be a safe and environmentally friendly approach. In addition, the exfoliated graphite shows high resveratrol adsorption capacity, which is ten times that of macroporous resins reported in the literature. Thus, the method proposed herein yields exfoliated graphite with high resveratrol adsorption capacity and is of great significance for the mass production of graphene for practical applications.

## 1. Introduction

Graphene, a honeycomb-structured two-dimensional material with C=C bonds, is formed from sp2-hybridized carbon atoms. Owing to its intriguing properties, graphene has shown broad application prospects in many fields. Although graphene was first prepared by Geim in 2004 [1], the commercial applications of graphene are still limited by a lack of processes for mass production.

Walimbe [2] proposes a sys-tematic methods which used for preparing graphene are generally classified as bottom-up and top-down methods. The bottom-up methods mainly include epitaxial growth on silicon carbide (SiC) [3,4,5] and chemical vapor deposition [6,7,8,9]. This approach can produce high-quality graphene sheets with perfect structure and large lateral area size but with higher production cost, lower production efficiency, and harsh reaction conditions, and it requires sophisticated equipment, greatly limiting its large-scale application. The top-down methods for the synthesis of graphene include mechanical exfoliation [1], solution exfoliation [10], ball milling [11,12], electrochemical methods [13,14,15], and oxidation–reduction methods. For instance, Geim [1] successfully fabricated graphene for the first time by mechanical exfoliation in 2004. Hou et al. [16] used expanded graphite as the raw material and prepared graphene sheets by a solvothermal exfoliation process, and its yield is about 10–12%. Although mechanical and solution exfoliation methods can yield graphene, their low graphene-stripping efficiency limits their widespread application. In 2013, Baek [17,18] mixed graphite with maleic anhydride or maleimide to obtain exfoliated graphite nanosheets using the ball milling method under solvent-free conditions. In 2016, He et al. [19] used oxone as an electrolyte to prepare graphene with 2–5 atomic layers via a one-step electrochemical method. In 2018, Sun et al. [20] proposed an electrochemical method to prepare exfoliated graphite at high yields using a ternary deep eutectic melt containing acetamide, urea, and ammonium nitrate. However, this method requires specific electrolytes and electrode materials, which is not conducive for commercial mass production. Until now, the oxidation–reduction method has been widely used for the preparation of graphene. Common graphite oxidation methods include the Brodie [21], Staudenmaier [22], and Hummers’ methods [23]. Although various modified versions of these methods have been reported, the requirement of strong acids and oxidants for these methods cannot be eliminated. Unfortunately, strong acids and oxidants destroy the main structure of graphene significantly and also produce a considerable amount of environmental pollutants, such as wastewater, waste acids, and unreacted strong oxidants. In summary, the current preparation methods of graphene have some shortcomings, and it is imperative to develop a facile and environmentally friendly method for the mass production of high-quality graphene.

The Diels–Alder reaction is one of the common chemical reactions involving conjugated alkenes. In 2012, Robert [24] made simulation calculations and found that graphene can be used as a diene or a dienophile in the Diels–Alder reaction due to its unique Dirac point structure. The finding was then confirmed by experiments [25]. Since then, several research studies proved that the ideal graphene edges could react with 2,3-dimethoxybutadiene, 9-methylanthracene, tetracyanoethylene, maleic anhydride, etc. through the Diels–Alder reaction [25,26]. Furthermore, the Diels–Alder reaction has been employed to chemically exfoliate graphite to obtain graphene and its analogs. In 2019, Tian [27] developed a method to exfoliate graphite in situ, in rubber and graphite blends, into graphene nanosheets based on the Diels–Alder reaction under strong shearing force. However, in the above methods, the exfoliation of graphite required strong shear force, and the thickness of the resultant exfoliated graphite was only reduced to the range of 0.1–1 μm. In the present study, the exfoliated graphite sample shows much smaller in thickness (about 0.5–1.5 nm) and could be obtained with the assistance of ultrasonic and without using any strong shearing force.

**Scheme 1:**
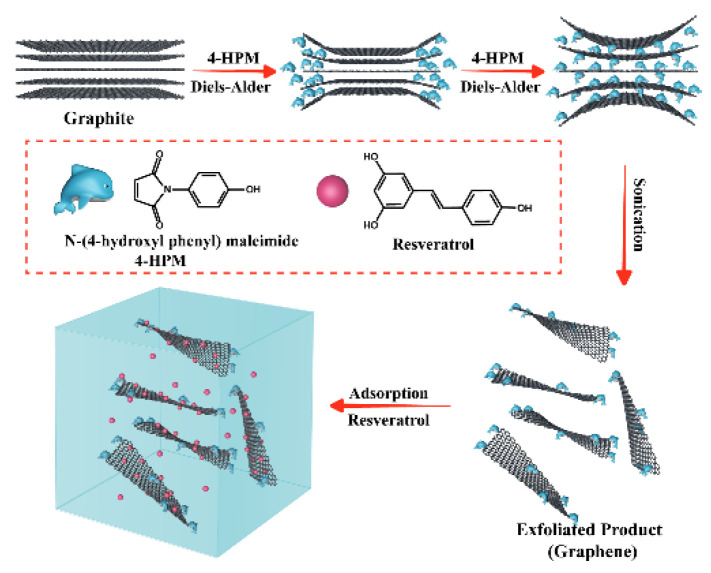
Schematic of the one-pot Diels–Alder reaction strategy for the preparation of graphene and resveratrol adsorption on the resultant graphene.

In this study, *N*-(4-hydroxyl phenyl) maleimide (4-HPM) was employed to exfoliate graphite into graphene. 4-HPM is a maleimide derivative with a bulk phenolic substituent on the N side, which may act as an effective molecular wedge to promote exfoliation when the C=C in the 4-HPM molecule undergoes the Diels–Alder reaction with the diene moiety of the graphite. In our previous work [28], the Diels–Alder reaction between graphene and 4-HPM was investigated intensively. Moreover, the phenolic hydroxyl group in the 4-HPM molecule also can improve the dispersibility and pH sensitivity of the functionalized graphene. Thus, a one-pot method was used to prepare graphene by exfoliating graphite using 4-HPM under heating and ultrasonic irradiating (Scheme 1). The as-prepared graphene was used for adsorbent of resveratrol, which was investigated as an example of plant extract medicines.

## 2. Materials and Methods

### 2.1. Materials

Graphite flakes (325 mesh, 99.8%) were purchased from Aladdin (Shanghai, China). Resveratrol (3,5,40-trihydroxy stilbene, ≥98%) and *N*-(4-hydroxyl phenyl) maleimide (4-HPM, 99.0%) were purchased from Heowns Company (Tianjin, China). N,N-dimethylformamide (DMF) (95.0%), *N*-methyl pyrrolidone (NMP) (95.0%), tetrahydrofuran (THF) (95.0%), p-xylene (PX) (95.0%), and absolute ethanol (EtOH) were purchased from Sinopharm Chemical Reagent Co. (Beijing, China) All the chemicals were used as received.

### 2.2. Exfoliation of Graphite via One-Step Diels–Alder Reaction

In a typical procedure, 40 mg of 4-HPM was dissolved in 40 mL of DMF in a flask followed by the addition of graphite (10 mg, G), and the mixture was sonicated for 30 min to obtain a homogeneous suspension. The suspension was heated at 90 °C in an oil bath for 60 min, followed by ultrasonication for 30 min at 250 W. Then, the resulting suspension was filtered using a polytetrafluoroethylene disc membrane (pore size = 0.1 μm and diameter = 50 mm), followed by several times washing with 100 mL DMF to remove any unreacted 4-HPM. The filtration residue was collected and dispersed in DMF by ultrasonication followed by centrifugation at 2000 rpm for 30 min. Then, the supernatant was dried to obtain exfoliated graphite compound (EGC).

### 2.3. Characterization

The microstructures and morphologies of graphite and the exfoliated graphite compound were examined by scanning electron microscopy (SEM) (JSM-6700F, operated at 5 kV) and atomic force microscopy (AFM) (Bruker-Multi Mode 8) (Akishima, Tokyo, Japan). The dispersion of samples in ethanol (0.1 mg/mL) was dropped on a silicon wafer to form a film by KW4A spin coater (Beijing SETCAS Electronics Co. Ltd. (Beijing, China) at 3000 r/min for 60 s. X-ray diffraction (XRD) of the samples was carried out on an X-ray diffractometer (Smartlab, Tokyo, Japan) with Cu-Kα radiation (wavelength = 0.15418 nm). Fourier-transform infrared (FT-IR) spectra of the samples were recorded on a Nicolet Nexus 470 IR spectrometer (FT-IR, Nicolet 6700, Austin, TX, USA) using the KBr co-grinding tablet method (sample:KBr = 1:100 in wt%; 32 scans were made for each spectrum). Raman spectroscopy was carried out on a Raman spectroscope (LabRAM HORIBA JY, Edison, NJ, USA) using a 785 nm laser. X-ray photoelectron spectroscopy (XPS) was carried out on a VG spectrometer (ESCALAB 250, Waltham, MA, USA) with focused monochromatic Al-Kα X-ray. The spectra were recorded at pass energies 200 eV and 30 eV for obtaining the survey and high-resolution spectra of the samples, respectively. The energy-dispersive X-ray spectroscope (EDS) images of the samples were obtained using a JSM-6700F instrument (JEOL, Tokyo, Japan).

## 3. Results and Discussion

### Characterization of Exfoliated Graphite Compound (EGC)

**Figure 1:**
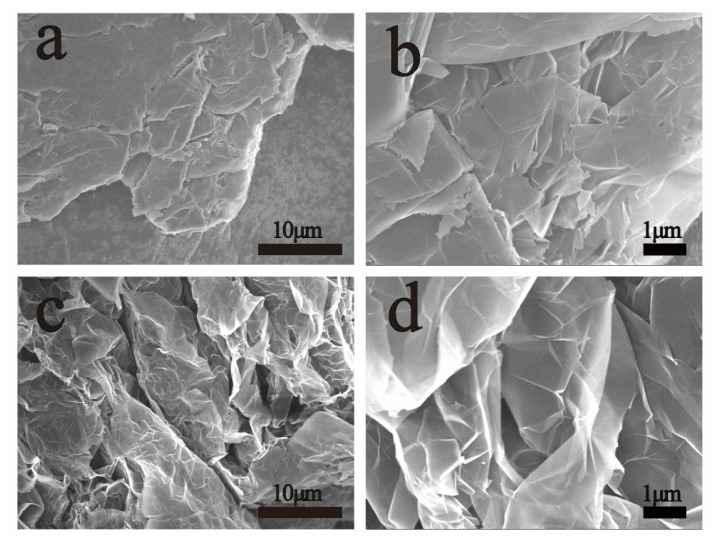
SEM images: (**a**) 10 μm light of the graphite; (**b**) 1 μm light of the graphite; (**c**) 10 μm light of the exfoliated graphite compound; (**d**) 1 μm light of the exfoliated graphite compound.

The microstructures of the graphite flakes and exfoliated graphite compounds were observed by SEM. The SEM images of the graphite flakes (Figure 1a) showed the presence of packed layers, which were flat and regular. However, these layers showed significant agglomeration, and the layer structure could hardly be detected. The exfoliated graphite compound obtained by the Diels–Alder reaction exhibited a fluffy sheet structure (Figure 1b), and the sheets were assembled similarly to a tulle. This structure is a typical microscopic feature of graphene sheets [17]. The significantly different microstructures between the graphite flakes and exfoliated graphite compound (Figure 1a,b) indicate that graphite was successfully exfoliated into graphene sheets via the Diels–Alder reaction.

**Figure 2:**
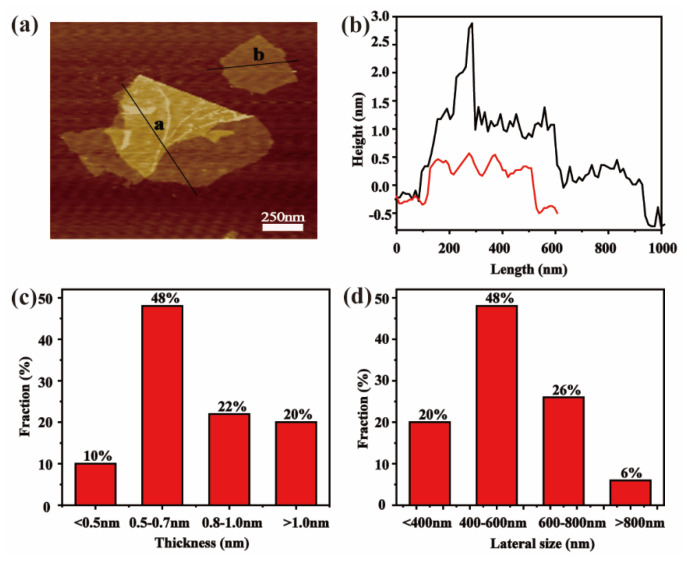
Characterization of exfoliated graphite compound by AFM: (**a**) AFM image and (**b**) the corresponding height profile; (**c**) statistical distribution of thickness and (**d**) lateral size distribution of exfoliated graphite compound obtained from the AFM images. The thickness and lateral size histograms were obtained from the analysis of 50 arbitrarily selected flakes.

To determine the thickness and lateral size of the exfoliated graphite compound, the flakes were transferred onto a Si substrate by spin coating for AFM measurements. The AFM image of exfoliated graphite compound is shown in Figure 2a. The exfoliated graphite compound consisted of large plates with a lateral size of ~500 nm and a thickness of ~0.7 nm (Figure 2b), corresponding to 1–2 layers (0.4–0.7 nm for a single layer in ref. [29]). To further demonstrate the efficiency of our preparation method, we arbitrarily selected and analyzed 50 flakes from the AFM images (Figure S1). The thickness and lateral size distributions of exfoliated graphite compounds are shown in Figure 2c,d. The statistical analysis of 50 randomly selected exfoliated graphite compound flakes revealed that 58% of exfoliated graphite compound sheets had a thickness of 0.4–0.7 nm, which corresponds to the thickness of one atomic layer. In addition, about 22% of the sheets had a thickness of 0.8–1.0 nm, which corresponds to two atomic layers of graphene. The lateral size statistical analysis (Figure 2d) revealed that more than 90% of exfoliated graphite compound sheets had a lateral dimension of 300–800 nm. Thus, our method yielded exfoliated graphite with a lateral size of about 300–800 nm and a thickness of 1–2 carbon atom layers. Compared with maleic anhydride intercalant [17,18], the graphene layers obtained by 4-HPM as intercalant are thinner, possibly because the molecular structure of 4-HPM is larger than that of maleic anhydride. The bulk phenolic substituent on the N site allows 4-HPM to act as a more effective molecular wedge to promote exfoliation.

**Figure 3:**
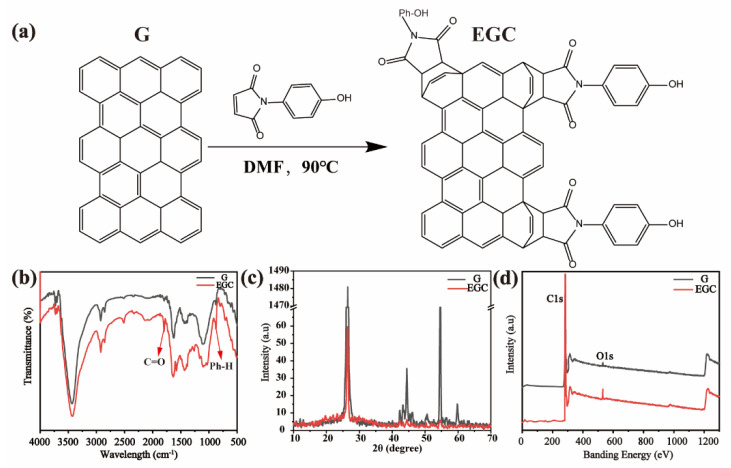
Scheme of reaction mechanism (**a**) and characterization of graphite/exfoliated graphite compound: (**b**) FT-IR spectra; (**c**) XRD patterns; (**d**) XPS profiles. G denotes graphite; EGC denotes exfoliated graphite compound.

The essence of the Diels–Alder reaction is that 4-HPM (as dienophile) and graphite sheet (as diene) are combined through [4+2] cycloaddition reaction (Figure 3a). The covalent grafting of 4-HPM molecules onto the exfoliated graphite compound surface was verified by FT-IR spectroscopy. Unlike graphite, the exfoliated graphite compound showed two peaks at 1798 and 875 cm^−1^, corresponding to the C=O stretching vibration and the bending vibration of hydrogen (Ph-H) in the benzene ring in 4-HPM [30]. Therefore, the FT-IR results confirms the occurrence of the Diels–Alder reaction between 4-HPM and graphite sheet. The crystal structures of graphite and exfoliated graphite compounds were analyzed by XRD (Figure 3c). It is observed that graphite has an intense characteristic peak at 26.5 degrees, corresponding to the 002 interplanar spacing of 3.36 Å [31]. The intensity of the diffraction peak corresponding to graphite (002, 2θ = 26.5°) decreased by several orders of magnitude in the case of exfoliated graphite compound. This is because the layers of exfoliated graphite compound became thinner as the Diels–Alder reaction progressed, and then the intensity of the corresponding XRD peaks decreased. This indicates that the Diels–Alder reaction decomposed the perfect crystal structure related to the c-axis in graphite, according to the method of Udayabhaskar [31] and Mohammadali [32]. The data of XRD (Table S1) were used to calculate the number of layers of exfoliated graphite compound. The calculated result shows that the thickness of the exfoliated product is equivalent to 1–3 layers of carbon sheet, which is consistent with the result of AFM (Figure 2).

XPS was employed to further analyze the elemental compositions of graphite and exfoliated graphite compounds. Figure 3d shows the survey spectra of graphite and exfoliated graphite compounds. The samples mainly consisted of carbon and oxygen. However, the oxygen peak intensity of exfoliated graphite compound was higher than that of graphite. Combined with the related analysis method about the graphene XPS spectra [33,34], we drew the spectrum of the N element (Figure S2c) and found that the characteristic peak (~298.2 eV) of the N element in the product is stronger than that in graphite. The O1s (Figure S2b) and N1s (Figure S2c) spectral results also are confirmed by EDS elemental maps (Figure S3). The XPS results confirmed that 4-HPM was involved in the exfoliation reaction.

**Figure 4:**
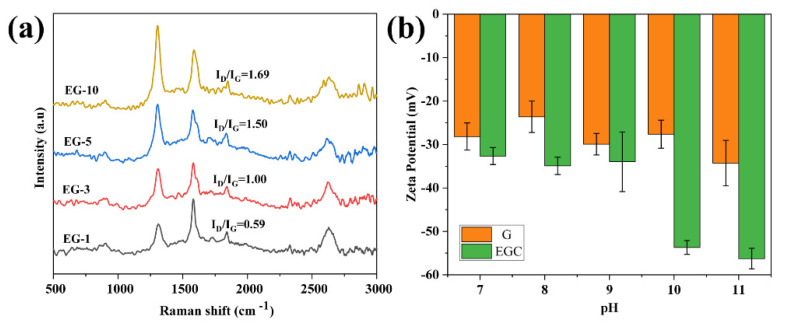
Raman spectra of samples with different heating–ultrasound times (**a**) and zeta potential of G/EGC (**b**). G denotes graphite; EGC denotes exfoliated graphite compound.

Ferrari reported that raman spectroscopy was the basic characterization technique for evaluating structural defects in graphene [35]. As shown in Figure 4a, the Raman spectra of graphite samples with different heating–ultrasound times all showed three peaks: a D peak at 1350 cm^−1^, a strong G peak at 1580 cm^−1^, and a 2D peak at 2708 cm^−1^, which were consistent with the data in previous reports [17,18,19,25,26]. In general, the intensity ratio of the D peak to G peak, I_D_/I_G_, of carbon material is used to determine the ratio of sp^3^ to sp^2^ carbon atoms, which is a measure of the number of defects in the material. As the reactions’ number increases, I_D_/I_G_ value (see Figure S4) also increases (from 0.59 to 1.69), which indicates that the sp^3^/sp^2^ carbon atom ratio increased and the degree of disorder of graphite increased after the Diels–Alder reaction. This can be attributed to the grafting of 4-HPM on the graphite during the heating–ultrasound process. Compared with only the ultrasound exfoliation method [10], the heating process (Diels–Alder reaction) has an obvious promotion effect. Therefore, the Diels–Alder reaction may be the driving force for the delamination of graphite into graphene nanosheets.

Figure 4b shows the zeta potential of graphite and exfoliated graphite compounds at different pH values. It is obvious that the zeta potential of graphite only fluctuates slightly with the change in pH. However, the zeta potential of exfoliated graphite sample negatively increased in the pH range of 10–11. Dissociation of the phenolic hydroxyl group in 4-HPM moiety is responsible for such zeta potential change. This result further confirms the success of the Diels–Alder reaction between graphite and 4-HPM. In addition, due to the introduction of the phenolic hydroxyl group from 4-HPM, the exfoliated product is functionalized, which exhibits better dispersion stability in common solvents (Figure S5). Our previous work has reported the dispersion stability of functionalized graphene [28].

The above results indicate that Diels–Alder reaction with 4-HPM can result in the exfoliation of graphite to yield exfoliated graphite. The yield of exfoliated graphite in this study is 38.0%, which is three times higher than that of the ultrasound exfoliation (12%) method in the ref. [10]. With the aid of ultrasound, 4-HPM enters between the graphite flakes as an intercalating agent and participates in the Diels–Alder reaction as a dienophile in the heating process. After multiple heating–ultrasonic irradiating processes, the interlayer spacing of graphite becomes larger, and finally, graphite is exfoliated into graphene. Compared with the conventional chemical exfoliation routes, the exfoliation process provided in the present study does not involve any acid or catalyst and could be carried out at a moderate temperature without vigorous stirring or shearing. Therefore, no water-washing and effluent treatment are required during the preparation of graphene. Our method may greatly reduce the consumption of water resources and alleviate the environmentally detrimental impact caused by the effluent of used chemicals. Moreover, the raw materials used in the present method, such as unexfoliated graphite and DMF, could be easily recycled; or, in a simple way, the unexfoliated graphite could continue the Diels–Alder reaction by adding new 4-HPM to improve the utilization of raw materials. Therefore, the method proposed in this study would be easily scalable.

To investigate the adsorption capacity, the exfoliated graphite was used as an adsorbent of resveratrol. The result showed that the resveratrol adsorption onto exfoliated graphite followed the pseudo-second-order kinetics, and the equilibrium data fitted well with the Langmuir isotherm model (Figure S6 and Table S2). Additionally, the adsorption behavior of exfoliated graphite for resveratrol was greatly affected with the pH value but little affected with the ionic strength (Figure S7). After 8 adsorbing–eluting cycles, the exfoliated graphite remains 80% of its adsorption capacity (Figure S8). Briefly, at 30 °C and pH 4, the exfoliated graphite showed a high resveratrol adsorption capacity (292.40 mg/g). Table 1 compares the adsorption capacity of the exfoliated graphite prepared in this study with those of the macroporous resins reported previously [36]. Clearly, the exfoliated graphite has higher resveratrol adsorption capacity (292.4 mg/g, 10 times higher) than the macroporous resins. The high resveratrol adsorption capacity (Figure S9) of the exfoliated graphite achieved in this study will be of great significance in the field of medicine.

## 4. Conclusions

In summary, an ultrasound-promoted one-pot Diels–Alder reaction strategy was developed to chemically exfoliate graphite into hydroxyl phenyl functionalized graphene using 4-HPM as a dienophile. The exfoliated graphite compound with a thickness of 1–3 layers of carbon skeleton was obtained. The as-obtained graphene had an average lateral size of about 500–800 nm. During the heating–ultrasonic irradiating process, 4-HPM acts as the intercalating agent, but also as the dienophile to participate in the Diels–Alder reaction. Consequently, the interlayer spacing of graphite grows and finally leads to exfoliation of graphite into phenolic functionalized graphene. On the one hand, the facile preparation method provided in this study does not require any acid or catalyst and has the potential to be amenable for mass production. On the other hand, the exfoliated graphite has excellent pH sensitivity owing to the introduction of 4-HPM. In addition, the exfoliated graphite shows high resveratrol adsorption capacity (292.4 mg/g), which is 10 times that of macroporous resins reported in the literature. Thus, the method proposed herein yields exfoliated graphite with high resveratrol adsorption capacity and is of great significance for the mass production of graphene for practical applications.

## Figures and Tables

**Table 1:** Resveratrol adsorption capacity of different adsorbents.

Adsorbent	Qmax, e (mg/g)	Adsorbent	Qmax, e (mg/g)
ADS-5	24.00	HPD-600	12.67
ADS-7	16.05	S-8	24.02
ADS-17	23.36	X-5	17.35
ADS-21	25.31	D101	14.68
NKA-9	20.65	Ab-8	22.67
NKA-11	18.00	Our work	292.40

## Data Availability

Our study did not report any data.

## Supplementary Materials

The following are available online at https://www.mdpi.com/article/10.3390/nano11113060/s1. Figure S1: Average thickness and lateral size obtained from the analysis of 50 sheets of exfoliated graphite compound, Figure S2: X-ray photoelectron spectral analysis of graphite and the exfoliated graphite compound: (a) survey spectra; (b) O1s spectra; (c) N1s spectra. G denotes graphite; EGC denotes exfoliated graphite compound, Figure S3: EDS elemental maps for (a) graphite and (b) exfoliated graphite compound. Figure S4: The calculation method of ID/IG ratio in Raman spectroscopy, Figure S5: Dispersions of exfoliated graphite in common solvents (0.5 mg/mL, after 24h), Figure S6: (a) Adsorption kinetics of the exfoliated graphite in an aqueous solution of resveratrol; (b) the pseu-do-first-order adsorption kinetics model; (c) the pseudo-second-order adsorption kinetics model; (d) resveratrol adsorption isotherm of the exfoliated graphite; (e) fitting with the Freundlich model; (f) fitting with the Langmuir model. (Temperature = 30 °C; pH = 4.0), Figure S7: Effect of solution pH (a) and ionic strength (b) on the adsorption of resveratrol onto exfoliated graphite (temperature = 30 °C), Figure S8: Recycling performance of exfoliated graphite (temperature = 30 °C; pH = 4.0; eluent: ethanol), Figure S9: UV–Vis spectra and fitting curve for resveratrol adsorption on the exfoliated graphite from solutions with different resveratrol concentrations, Table S1: Structural parameters of the exfoliated graphite from the XRD data, Table S2: Kinetic parameters and isotherm fitting parameters for the adsorption of resveratrol on the exfoliated graphene.
