# Supplementary material for: Ultrasonic-Assisted Diels–Alder Reaction Exfoliation of Graphite into Graphene with High Resveratrol Adsorption Capacity
Source: Nanomaterials (Basel). 2021 Nov 14;11(11):3060. doi: 10.3390/nano11113060 (PMC8623989; doi:10.3390/nano11113060)
Supplement: Supplementary file 1 [file nanomaterials-11-03060-s001.zip › nanomaterials-1465480-supplementary.pdf]

Supplementary Materials

Ultrasonic-Assisted Diels–Alder Reaction Exfoliation of Graphite into Graphene with High Resveratrol Adsorption Capacity

Jinxing Zhang †, Qi Ouyang †, Qilin Gui *, and Xiaonong Chen *

Beijing Laboratory of Biomedical Materials, Beijing University of Chemical Technology, Beijing 100029, China; zhangjinxing1202@163.com (J.Z.); ouyangqi0000@163.com (Q.O.)

* Correspondence: guiql@mail.buct.edu.cn (Q.G.); chenxn@mail.buct.edu.cn (X.C.)

† These authors contributed equally to this work.

Experimental section of adsorption

1. Adsorption Kinetics

To analyze the adsorption kinetics, the exfoliated graphene (20.0 mg) was mixed with 100 mL of a resveratrol aqueous solution (100 mg/L, pH = 4.0, 30 °C). The resulting mixture was shaken for different time intervals (5–300 min). The exfoliated graphene and resveratrol aqueous solution were then separated. The concentration of resveratrol was determined from its absorption measured using a UV-2550 spectrophotometer at the wavelength of 290 nm. The resveratrol concentration (C_t) was calculated using the calibration curve (Figure S9), and the adsorption capacity (q_t) was calculated using the formula: $(C_0–C_t)V/m_{FG}$. The data was analyzed by pseudo-first-order (Equation S1) and pseudo-second-order (Equation S2) models as follows:

$$\mathrm{Ln}(q_e - q_t) = \mathrm{Ln}q_e - k_1\mathrm{t} \tag{S1}$$

$$\frac{t}{q_t} = \frac{1}{k_2 q_e^2} + \frac{t}{q_e} \tag{S2}$$

where q_t (mg/g) is the amount of resveratrol adsorbed at different time intervals, q_e (mg/g) is the amount of resveratrol adsorbed at equilibrium time, and k_1 (min^{-1}) and k_2 (min·g·mg^{-1}) are the rate constants of the pseudo-first-order and pseudo-second-order kinetic models, respectively.

2. Adsorption Isotherms

For obtaining the isotherms of resveratrol adsorption onto exfoliated graphene, the initial pH of the resveratrol solution was maintained at 4. The concentration of exfoliated graphene was 0.5 mg/mL, while the resveratrol concentration was varied from 10 to 100 mg/L. After shaking for 400 min, the exfoliated graphene was removed from the solution by filtration and the concentration of resveratrol in the filtrate was analyzed according to the Freundlich (Equation S3) and Langmuir adsorption (Equation S4) equations:

$$Lnq_e = LnK_F + \frac{1}{n}LnC_e \tag{S3}$$

$$\frac{C_e}{q_e} = \frac{1}{K_L q_{max}} + \frac{C_e}{q_{max}} \tag{S4}$$

where q_{max} is the maximum adsorption (mg/g), q_e is the amount of resveratrol adsorbed on the exfoliated graphene at equilibrium (mg/g), C_e is the resveratrol concentration in the solution at equilibrium (mg/L), and K_F and 1/n are the Freundlich characteristic constants, representing the adsorption capacity and adsorption intensity, respectively. K_L is the Langmuir adsorption equilibrium constant (mg/L) representing the energy of adsorption.

3. Effect of pH and Ionic Strength on the Resveratrol Adsorption Capacity of Exfoliated Graphene

The effect of the resveratrol solution pH on the resveratrol adsorption of the exfoliated graphene was investigated by varying the pH of the solution from 3.0 to 9.0. A contact time of 400 min was used. The pH of the solution was adjusted by adding aqueous solutions of 0.01 mol/L HCl or 0.01 mol/L NaOH to it.

The effect of the ionic strength of the resveratrol solution on the resveratrol adsorption of the exfoliated graphene was investigated by adding sodium chloride to the solution and varying its concentration from 0 to 0.1 mol/L.

4. Regeneration of Exfoliated Graphene

For regeneration, the exfoliated graphene after adsorbing resveratrol was washed with ethanol followed by filtering (using PTFE disc membrane with 0.1 μm pore size). Then the sample was lyophilized. The resveratrol adsorption capacity of the dried sample was then evaluated. This adsorption-desorption cycle was repeated eight times to evaluate the regeneration performance of the exfoliated graphene.

Result Section

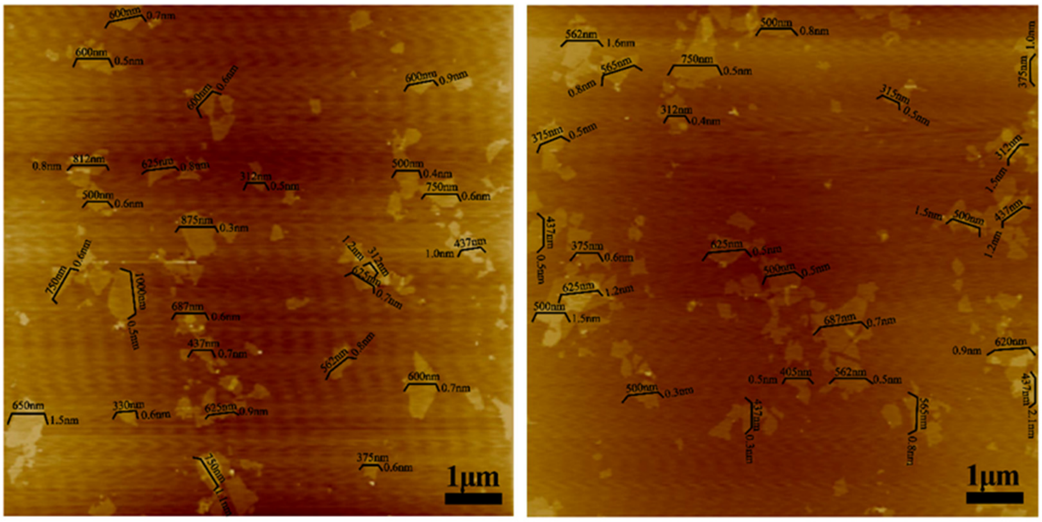

Figure S1. Average thickness and lateral size obtained from the analysis of 50 sheets of exfoliated graphite compound.

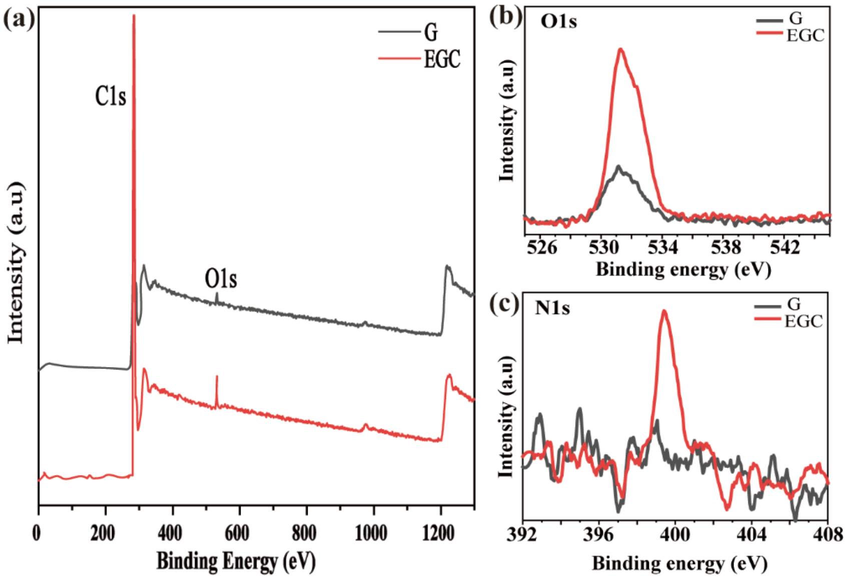

Figure S2. X-ray photoelectron spectral analysis of graphite and the exfoliated graphite compound: (**a**) survey spectra; (**b**) O1s spectra; (**c**) N1s spectra. G denotes graphite; EGC denotes exfoliated graphite compound.

Table S1. Structural parameters of the exfoliated graphite from the XRD data.

Sample	2θ (deg)	FWHM*	γ (nm)	d (nm)	N
Exfoliated graphite	26.42	0.54	0.154	0.26	1~2

*FWHM: Full Width at Half Maxima of the exfoliated graphite.

The values of average height (γ (nm)), average number of graphite layers (N) and interlayer spacing (d (nm)) of the graphene stacking layers are provided for comparison.

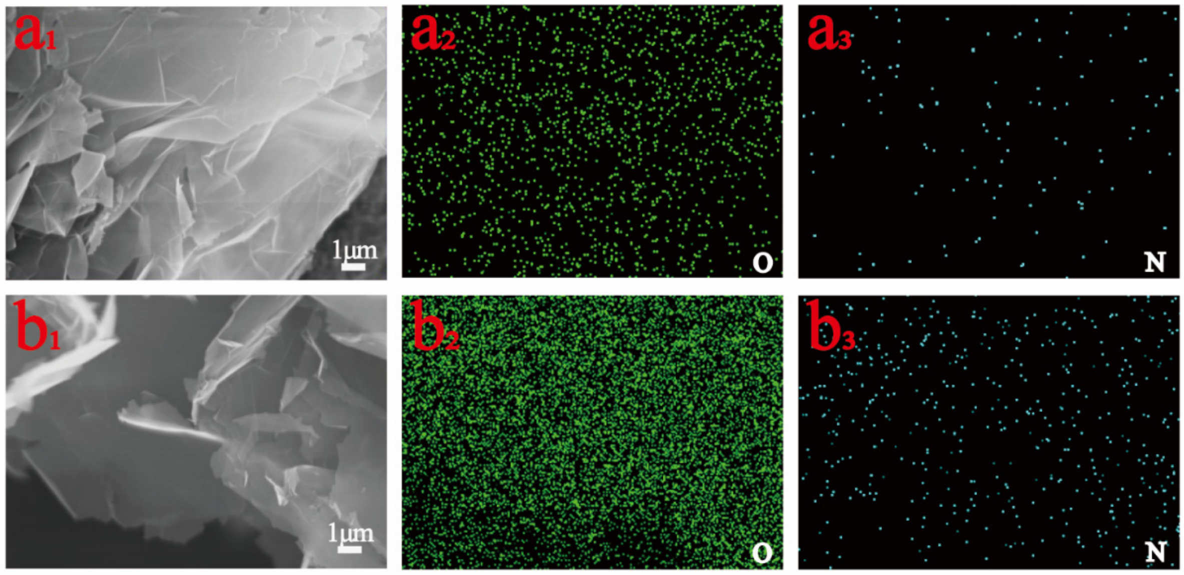

Figure S3. EDS elemental maps for (**a**) graphite and (**b**) exfoliated graphene.

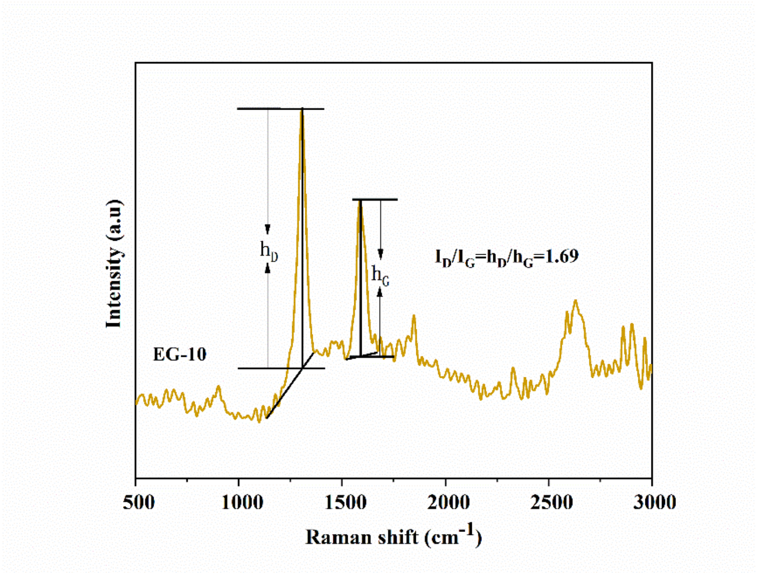

Figure S4. The calculation method of I_D/I_G ratio in Raman spectroscopy.

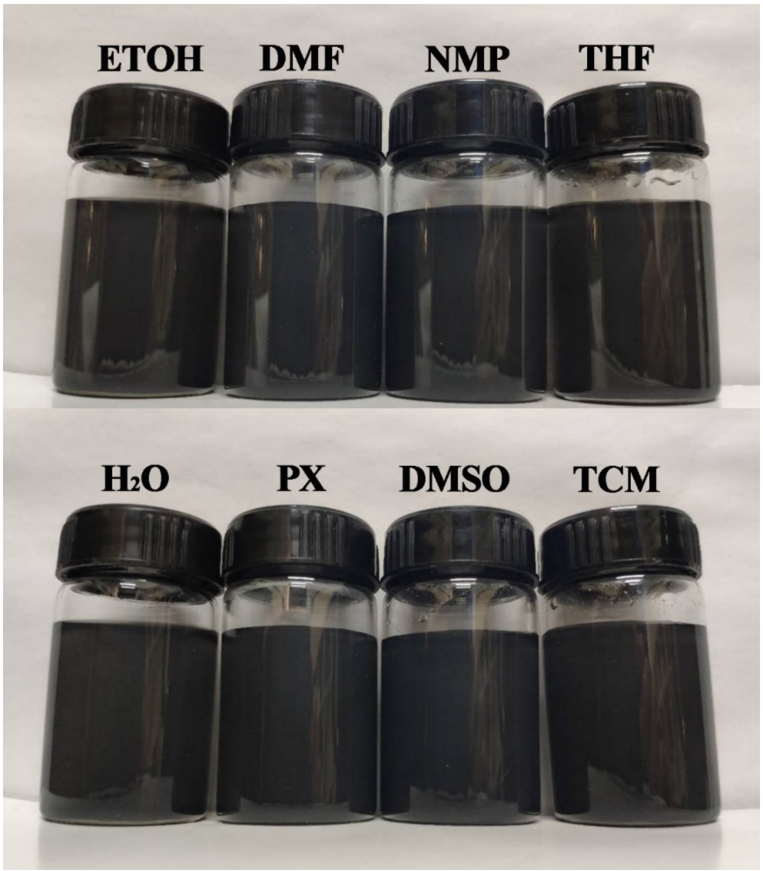

Figure S5. Dispersions of exfoliated graphite in common solvents (0.5 mg/mL, after 24h).

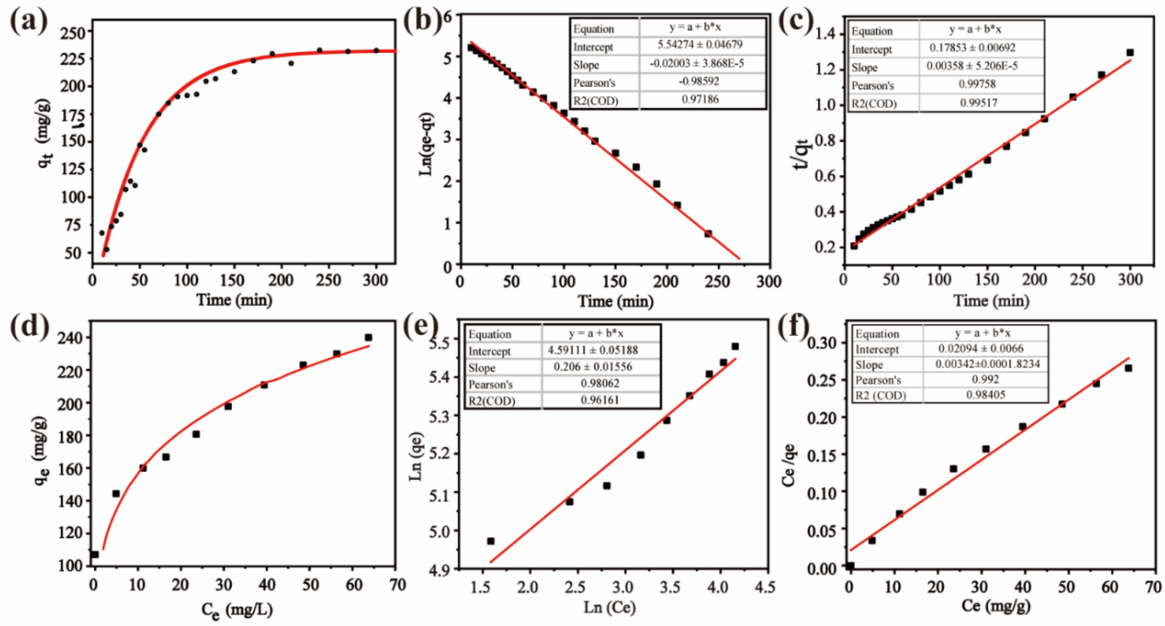

Figure S6. (**a**) Adsorption kinetics of the exfoliated graphene in an aqueous solution of resveratrol. (**b**) The pseudo-first-order adsorption kinetics model. (**c**) The pseudo-second-order adsorption kinetics model. (**d**) Resveratrol adsorption isotherm of the exfoliated graphene. (**e**) Fitting with the Freundlich model. (**f**) Fitting with the Langmuir model. (Temperature = 30 °C; pH = 4.0).

Table S2. Kinetic parameters and isotherm fitting parameters for the adsorption of resveratrol on the exfoliated graphene.

Kinetic model	Pseudo-first-order model				Pseudo-second-order model			
	$q_{e,exp}$ (mg/g)	K_1	$q_{e,cal}$ (mg/g)	R^2	$q_{e,exp}$ (mg/g)	K_2	$q_{e,cal}$ (mg/g)	R^2
	232.27	0.020	255.377	0.972	232.27	7.2×10^{-5}	279.33	0.995

Isotherm data	Freundlich model			Langmuir model		
	1/n	K_F (L/g)	R^2	q_{max} (mg/g)	K_L (L/g)	R^2
	0.206	98.604	0.962	292.400	0.163	0.984

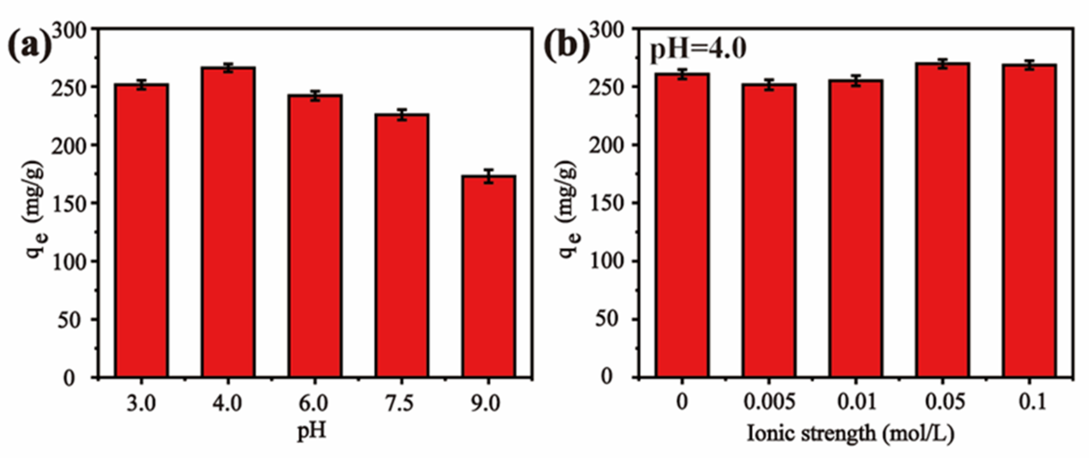

Figure S7. Effect of solution pH (**a**) and ionic strength (**b**) on the adsorption of resveratrol onto exfoliated graphene (Temperature = 30 °C).

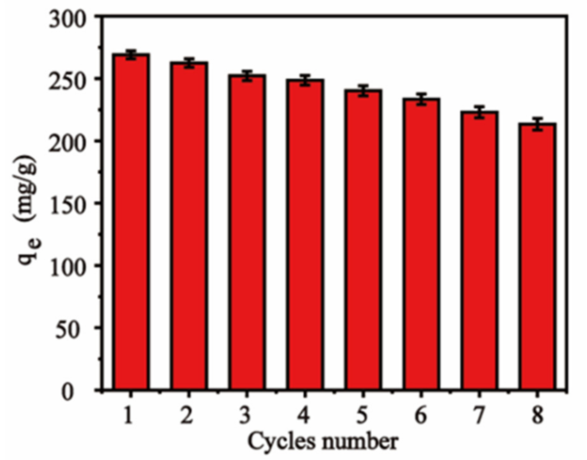

Figure S8. Recycling performance of exfoliated graphene (Temperature = 30 °C; pH = 4.0; Eluent: ethanol).

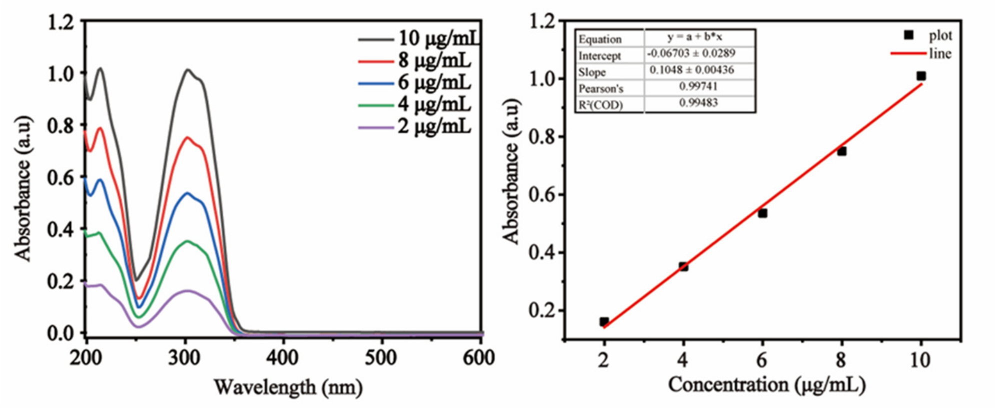

Figure S9. UV-vis spectra and fitting curve for resveratrol adsorption on the exfoliated graphene from solutions with different resveratrol concentrations.
